# Comparison of the pathogenicity of a Taiwan lineage 1 NADC30/34-like recombinant and a lineage 3 *Betaarterivirus suid 2* in pigs

**DOI:** 10.3389/fvets.2026.1969559

**Published:** 2026-10-27

**Authors:** Yi-Fang Lai, Chao-Nan Lin, Ming-Tang Chiou, Yen-Chen Chang, Hui-Wen Chang

**Affiliations:** 1 Department of Veterinary Medicine, School of Veterinary Medicine, National Taiwan University, Taipei, Taiwan; 2 Animal Disease Diagnostic Center, College of Veterinary Medicine, National Pingtung University of Science and Technology, Pingtung, Taiwan; 3 Department of Veterinary Medicine, College of Veterinary Medicine, National Pingtung University of Science and Technology, Pingtung, Taiwan; 4 School of Veterinary Medicine, Graduate Institute of Molecular and Comparative Pathobiology, National Taiwan University, Taipei, Taiwan

**Keywords:** *Betaarterivirus suid 2*, cytokines, lineage 1, lineage 3, pathogenicity, Taiwan

## Abstract

Porcine reproductive and respiratory syndrome virus 2 (PRRSV-2; *Betaarterivirus suid 2*) causes porcine reproductive and respiratory syndrome, leading to severe economic losses in the global swine industry. Lineage 1 (L1) PRRSV-2 has emerged and become increasingly prevalent in North America and Asia. This study compared the pathogenic characteristics of a Taiwan NADC30/34-like L1 recombinant and a lineage 3 (L3) PRRSV-2 strain and evaluated their suitability for establishing an experimental infection model in growing pigs. Ten-week-old pigs were inoculated with either the NADC30/34-like L1 NPUST-107-844/2018 strain or the L3 SW2018001-YL strain via concurrent intranasal and intramuscular routes. Pigs inoculated with the L1 strain exhibited higher body temperatures and a greater frequency of fever during the early post-inoculation period than those inoculated with the L3 strain. However, no significant differences were observed between the groups in peak viremia, lung pathological scores, or plasma cytokine concentrations. These results indicate that the Taiwan L1 strain induced a more pronounced early febrile response but produced virological, pulmonary pathological, and systemic cytokine responses comparable to those induced by the Taiwan L3 strain. Collectively, these findings highlight the pathogenic diversity of circulating PRRSV-2 strains in Taiwan and for future experimental studies, including investigations of disease pathogenesis, therapeutic interventions, and vaccine development.

## 1 Introduction

Porcine reproductive and respiratory syndrome (PRRS) is a major global disease affecting the swine industry that causes severe economic loss. The clinical symptoms of PRRS may vary from asymptomatic to devastating disease, including respiratory and reproductive symptoms, reduced feed efficiency, and increased mortality (1).

Porcine reproductive and respiratory syndrome (PRRS) is caused by porcine reproductive and respiratory syndrome virus 1 (PRRSV-1) and PRRSV-2, which are classified within the species *Betaarterivirus suid 1* and *Betaarterivirus suid 2*, respectively, in the family *Arteriviridae* (2). It is a single-stranded positive-sense RNA virus. The overall genome size of PRRSV is approximately 14.9 to 15.5 kb in length and comprises 11 open reading frames (ORFs) (3). ORF1a is translated into polyprotein pp1a, whereas a programmed ribosomal frameshift allows translation to continue through ORF1b, producing polyprotein pp1ab. These polyproteins are proteolytically processed into nonstructural proteins involved in viral replication. The minor envelope glycoproteins, including glycoprotein 2 (GP2), E, GP3, and GP4, were encoded in ORF2a, ORF2b, ORF3, and ORF4, respectively. The major envelope glycoproteins GP5, membrane protein (M), and nucleocapsid (N), encoded by ORF5, ORF6, and ORF7, respectively, are related to immunogenicity (4–8).

PRRSV-1 and PRRSV-2 were historically referred to as the European and North American genotypes, respectively, but are currently classified as viruses belonging to two distinct species (9). In Taiwan, the first PRRSV outbreak was identified in 1991 and was attributed to the PRRSV-2 group. From 2004 to 2006, a study showed that most of Taiwan's PRRSV strains belong to PRRSV-2 lineage (L)3, with only a few cases reported as PRRSV-2 L5 vaccine-related strains. From 2014 to 2017, most of the Taiwan PRRSV strains belonged to L3 (10). In March 2018, the first NADC-like strain, designated PRRSV/TW/NPUST-107-844/2018 (NPUST-107-844/2018), causing an abortion storm, was identified (11). These PRRSV-2 NADC-like L1 strains were suspected as circulating recombinants of NADC30/34-like L1 strains based on high sequence identity to the NADC34 strain in ORF2-5, the detection of unique a deletions identical to NADC30, and the high sequence identity to the NADC30 strain in the Nsp2 gene (11). Since then, the prevalence of the NADC-like L1 strains increased to as high as 47% in Taiwan (12–14). Several outbreaks of high mortality and abortion rates in sows are suggested to be associated with PRRSV-2 NADC-like L1 strains in Taiwan (13). Furthermore, molecular evidence suggests that some PRRSV-2 NADC-like L1 strains may be recombinants involving Taiwan L3 strains (14).

Using neonatal piglets would require maintaining piglets with their sows throughout the experimental period, substantially increasing housing, feeding, labor, and animal-management costs. PRRSV can infect pigs of different ages in the absence of protective immunity (15). Establishing a challenge model in growing pigs provides a practical, reproducible, and clinically relevant platform for preliminarily evaluating PRRSV vaccines and other intervention strategies (16–18) and is particularly suitable for vaccines requiring multiple-dose immunization. Thus, this model offers a cost-effective, manageable, and field-relevant platform for assessing PRRSV vaccines and intervention strategies.

This study aimed to compare the pathogenicity of the emerging Taiwan PRRSV-2 NADC30/34-like L1 (the NADC-like L1) recombinant with the PRRSV-2 L3, and to establish standardized viral experimental infection models in growing pigs. Clinical outcomes, including viremia, body weight changes, and clinical signs, were monitored, and gross and histopathological lesions were evaluated. Through comparative analysis, this study sought to clarify differences in virulence and disease progression between L1 and L3 PRRSV-2 strains and to provide an experimental basis for understanding the pathogenic characteristics of currently circulating PRRSV variants in Taiwan.

## 2 Materials and methods

### 2.1 Cell line and viruses

MARC-145 (ATCC No.: CRL-12231) was maintained in Dulbecco's Modified Eagle Medium (DMEM; Thermo Fisher Scientific Inc., Waltham, MA, USA) supplemented with 10% FBS (Millipore, Burlington, MA, USA) and penicillin/streptomycin sulfate (Thermo Fisher Scientific Inc.). Porcine alveolar macrophages (PAMs) were collected by bronchoalveolar lavage as previously described (19) and cultured in RPMI-1640 medium (Thermo Fisher Scientific Inc.) supplemented with 10% FBS and penicillin/streptomycin sulfate. All culture incubations were performed in a humidified 37 °C, 5% CO_2_ incubator (Thermo Fisher Scientific Inc.).

The viruses used in the present study were the Taiwan NADC-like L1 strain, NPUST-107-844/2018 (GenBank accession no. MK291410.1) (11), and the Taiwan L3 strain, SW2018001-YL (GenBank accession no. MN401750.1). NPUST-107-844/2018 was isolated from an aborted fetus and propagated in PAMs. The strain SW2018001-YL, which belongs to the PRRSV-2 L3, was isolated from a postweaning pig in 2018 and adapted to MARC-145 cells. At each passage, cultures were harvested when approximately 80% cytopathic effect was observed. The cultures were frozen at −80 °C and thawed once, and the resulting culture fluid was passed through a 0.2-μm filter. The filtered supernatant was subsequently used to inoculate cells for the next passage. Both viruses were used at passage 6 for preparation of the challenge stocks. The viral titer of the two challenge stocks was 10^4^ TCID_50_/mL, and both viral stocks were tested for the absence of porcine circovirus type 2, swine influenza A virus, and pseudorabies virus.

### 2.2 Animal experimental infection

Eight nine-week-old, specific pathogen-free pigs purchased from the Agricultural Technology Research Institute (Hsinchu, Taiwan) tested negative for PCV2 and PRRSV by real-time PCR. The pigs were individually identified using ear tags and randomly allocated to either the SW2018001-YL (Taiwan L3) inoculation group (*n* = 4) or the NPUST-107-844/2018 (Taiwan NADC-like L1) inoculation group (*n* = 4). At 10 weeks of age, each pig was inoculated with the corresponding virus through concurrent intramuscular and intranasal routes. One milliliter of virus suspension containing 10^4^ TCID_50_ was administered intramuscularly, and an additional 1 mL containing 10^4^ TCID_50_ was administered intranasally, resulting in a total inoculation dose of 2 × 10^4^ TCID_50_ per pig. The body temperature and clinical signs were monitored daily. Body temperature was measured using an RFID temperature-sensing microchip (UID Temperature Programmable Microchip UCT-2112; Unified Information Devices, Lake Villa, IL, USA) implanted subcutaneously in the postauricular region. Temperature readings were obtained by scanning the microchip using a compatible RFID reader. Fever was defined as a body temperature of ≥40.0 °C. Ten mL of ethylenediaminetetraacetic acid (EDTA)-anticoagulated blood was collected at 3 days post-inoculation (DPI) and every week for 3 weeks to detect viremia, cytokine profile, and PRRSV-specific plasma IgG and cellular immunity. At 3 weeks post-inoculation (WPI), all pigs were humanely euthanized for pathological evaluation of lesions. Animals were euthanized in accordance with the Council of Agriculture, Executive Yuan, Guidelines for the Care and Use of Laboratory Animals. Animals were first be effectively stunned by electrical stunning using electrodes placed bilaterally on the head at a voltage of at least 220 V for 10 s. Death was then induced by severing major arteries and exsanguination within 15 s after stunning. All experimental procedures performed on the animals were reviewed and approved by the Institutional Animal Care and Use Committee of National Taiwan University (Taipei, Taiwan, NTU113-0085).

### 2.3 Viral detection

Nucleic acids were extracted from plasma and homogenized tissues using the Maelstrom^*TM*^ 4810 automated nucleic acid extractor (Taiwan Advanced Nanotech, Taoyuan, Taiwan). PRRSV RNA loads in plasma and tissue samples were quantified using the QuantiTect Probe RT-PCR Kit (Qiagen, Hilden, Germany) with primers and a ZNA probe targeting the PRRSV ORF6 gene, as described previously with modifications (20). The one-step real-time RT-PCR cycling conditions consisted of reverse transcription at 50 °C for 30 min, initial PCR activation at 95 °C for 15 min, followed by 40 cycles of denaturation at 95 °C for 15 s, annealing/extension at 60 °C for 60 s, with fluorescence acquisition at the end of each annealing/extension step.

Plasma PRRSV RNA loads were calculated according to the volume of plasma used for nucleic acid extraction, the nucleic acid elution volume, and the volume of template added to each RT-qPCR reaction. Results were expressed as log10 PRRSV RNA copies per milliliter of plasma.

For tissue samples, porcine GAPDH RNA was quantified using the QuantiTect Probe RT-PCR Kit (Qiagen) with the primers and probe shown in Table 1. The cycling conditions consisted of reverse transcription at 50 °C for 30 min, initial PCR activation at 95 °C for 15 min, followed by 40 cycles of denaturation at 95 °C for 15 s, annealing/extension at 55 °C for 60 s, with fluorescence acquisition at the end of each annealing/extension step. Tissue PRRSV RNA levels were normalized to the relative GAPDH abundance to account for variations in host RNA input. For each tissue, the mean GAPDH Ct value across all samples was used as the reference. Tissue PRRSV RNA levels were analyzed using two-way ANOVA with tissue and inoculation group as factors, followed by Šídák's multiple-comparisons test. The primers and probes used for the PRRSV and GAPDH assays are listed in Table 1.

**Table 1:** . Primers and probes used for real-time RT-PCR quantification of PRRSV-2 and porcine GAPDH.

Primer or probe	Sequence (5′-3′)	Reference
PRRSV-M177F	CATTCTGGCCCCTGCCCA	(20)
PRRSV-M177R	ACCACTCCYYGYTTDACAGCT	
PRRSV-II probe	FAM-CTCGTGTTGGGTGGCAGA-ZNA4-BHQ1	
GAPDH-F	ACATGGCCTCCAAGGAGTAAGA	(43)
GAPDH-R	GATCGAGTTGGGGCTGTGACT	
GAPDH probe	Texas Red-CCACCAACCCCAGCAAGAGCACGC-BHQ2	

ZNA, zip nucleic acid; FAM, 6-carboxyfluorescein; BHQ, Black Hole Quencher; GAPDH, glyceraldehyde-3-phosphate dehydrogenase.

To confirm the primers and probe suitability for both challenge viruses, the forward primer, reverse primer, and probe sequences were aligned with the corresponding ORF6 sequences of the SW2018001 and NPUST-107-844/2018 isolates. The primer- and probe-binding regions were fully conserved in both isolates, with the degenerate nucleotides in the reverse primer accommodating the sequence variation between the two strains. No mismatches were identified within any of the primer- or probe-binding regions. Because the same primer/probe set, reaction conditions, and standard curve were applied consistently to all samples, the assay was considered appropriate for detecting and comparing PRRSV RNA levels between the two inoculation groups.

### 2.4 Determination of cellular responses by enzyme-linked immunospot (ELISPOT)

Peripheral blood mononuclear cells (PBMCs) were isolated from EDTA-anticoagulated blood by density-gradient centrifugation using Ficoll-Paque™ PLUS (Cytiva, Marlborough, MA, USA). The buffy coat was diluted with RPMI-1640 medium to a final volume of 7 mL, carefully layered onto Ficoll-Paque PLUS, and centrifuged at 3,000 rpm for 30 min at 20 °C with the centrifuge brake off. Residual erythrocytes were removed using ACK lysis buffer. The isolated PBMCs were washed, resuspended in culture medium, and assessed for viability using the trypan blue exclusion assay. PRRSV-specific IFN-γ-secreting cells were quantified using a porcine IFN-γ single-color ELISPOT kit (ImmunoSpot^®^, Cellular Technology Limited, Cleveland, OH, USA) according to the manufacturer's instructions. PBMCs were seeded at 3 × 105 cells per well and stimulated with 200 TCID_50_ per well of either the Taiwan NADC-like L1 strain NPUST-107-844/2018 or the Taiwan L3 strain SW2018001-YL for 24 h. PBMCs stimulated with concanavalin A (0.5 μg/mL) served as positive controls, whereas PBMCs incubated with CTL medium without viral antigen served as negative controls. ELISPOT plates were analyzed using a Cytation 7 multimode imager with Gen5 software (Agilent Technologies, Santa Clara, CA, USA). PRRSV-specific IFN-γ-secreting cell counts were calculated by subtracting the mean spot count of the corresponding unstimulated control wells from that of the virus-stimulated wells. Results were expressed as the number of IFN-γ-secreting cells per 106 PBMCs.

### 2.5 Antibody analysis

PRRSV-specific antibodies in plasma were detected using the IDEXX PRRS X3 Ab Test (IDEXX Laboratories, Westbrook, ME, USA) according to the manufacturer's instructions. Samples with a sample-to-positive ratio of ≥0.4 were considered seropositive.

### 2.6 Cytokine analysis

Commercial ELISA assays were used to determine the post-inoculation induction of cytokines, including TNF-α (Abcam Limited, Cambridge, UK), IL-1β (Abcam Limited, Cambridge, UK), IL-4 (Thermo Fisher Scientific, Waltham, MA, USA), and IL-10 (Thermo Fisher Scientific, Waltham, MA, USA). The manufacturer's guidelines were followed during the performance of the ELISA test. Samples were analyzed in single wells, and cytokine concentrations were calculated from the corresponding standard curves.

### 2.7 Pathological evaluation

At three WPI, all pigs were euthanized for pathological examination. All euthanasia procedures were performed in accordance with the AVMA Guidelines for the Euthanasia of Animals: 2020 Edition (American Veterinary Medical Association, Schaumburg, IL, USA). At necropsy, the percentage of grossly affected tissue was estimated separately for each lobe. A weighted total gross lung lesion score ranging from 0% to 100% was calculated. The percentage of affected tissue in each lobe was multiplied by its corresponding weighting factor, and the weighted values were summed to obtain the total percentage of affected lung (21). Following gross examination, representative lung samples were collected from each lobe, fixed in 10% neutral-buffered formalin, routinely processed, embedded in paraffin, sectioned at 4 μm, and stained with hematoxylin and eosin. Microscopic pulmonary lesions were evaluated for the severity and distribution of interstitial pneumonia using a scale from 0 to 6: 0, normal; 1, mild multifocal; 2, mild diffuse; 3, moderate multifocal; 4, moderate diffuse; 5, severe multifocal; and 6, severe diffuse (21). Gross and microscopic pulmonary lesions were evaluated by an observer blinded to the inoculation groups.

### 2.8 Immunohistochemistry (IHC) staining

Paraffin-embedded lung sections (4 μm) were deparaffinized in xylene and rehydrated through graded ethanol. Antigen retrieval was performed using Trilogy™ solution at 106–110 °C for 15 min in a pressure cooker. Endogenous peroxidase activity was blocked using 3% hydrogen peroxide in methanol for 15 min. After blocking with 10% normal goat serum in phosphate-buffered saline for 30 min at room temperature, the sections were incubated with a mouse monoclonal antibody against the PRRSV nucleocapsid protein (clone 1AC7; INGENASA, Madrid, Spain; 1:800) for 1 h at room temperature. After washing with TBST, the sections were incubated with HRP-conjugated goat anti-mouse IgG (H+L) (Jackson ImmunoResearch Laboratories, West Grove, PA, USA; 1:200) for 60 min at room temperature. Immunoreactivity was visualized using DAB+ chromogen (Dako, Glostrup, Denmark), and the sections were counterstained with hematoxylin for 30 s. Negative-control sections were processed identically, except that the primary antibody was omitted. PRRSV antigen immunoreactivity was semiquantitatively scored using a four-grade system: grade 0, no positive cells; grade 1, 1–20 positive cells in the entire section; grade 2, positive cells occupying ≤ 50% of one high-power field; and grade 3, positive cells occupying >50% of one high-power field or ≥30 positive cells in at least two to three high-power fields (22).

### 2.9 Statistics

Statistical analyses and data visualization were performed using GraphPad Prism version 11.0.2 (GraphPad Software, San Diego, CA, USA). Data are presented as the mean ± standard deviation (SD). Repeated measurements, including body temperature, body weight, plasma viral RNA load, antibody responses, PRRSV-specific IFN-γ-secreting cell responses, and plasma cytokine concentrations, were analyzed using two-way repeated-measures analysis of variance (ANOVA), followed by Šídák's multiple-comparisons test. When missing values prevented repeated-measures ANOVA, a mixed-effects model fitted by restricted maximum likelihood was used, followed by Šídák's multiple-comparisons test. Gross lung lesion percentages were compared using an unpaired two-tailed *t-test*. Microscopic lung lesion scores and IHC scores were compared using a two-tailed Mann–Whitney test. A *P*-value of less than 0.05 was considered significant.

## 3 Results

### 3.1 The clinical signs, body weight, and body temperature

Following viral inoculation, depression and reduced appetite were observed in both groups. The L1-inoculated group generally showed higher mean body temperatures than the L3-inoculated group during the observation period (Figure 1A). In the L1-inoculated group, fever was detected in one pig at 1 DPI, two to four pigs between 3 and 9 DPI, all four pigs at 12 DPI, and three pigs at 23 DPI (Figure 1B). In comparison, only one or two pigs in the L3-inoculated group developed fever during the study period. Although no statistically significant between-group difference in body weight was detected during the experimental period (Figure 1C), analysis of average daily weight gain revealed a significant reduction in the L3-inoculated group compared with the L1-inoculated group during the early post-inoculation period from 0 to 7 DPI (Figure 1D). No statistically significant between-group differences in average daily weight gain were detected during the pre-inoculation period from −7 to 0 DPI or the later period from 7 to 21 DPI.

**Figure 1:**
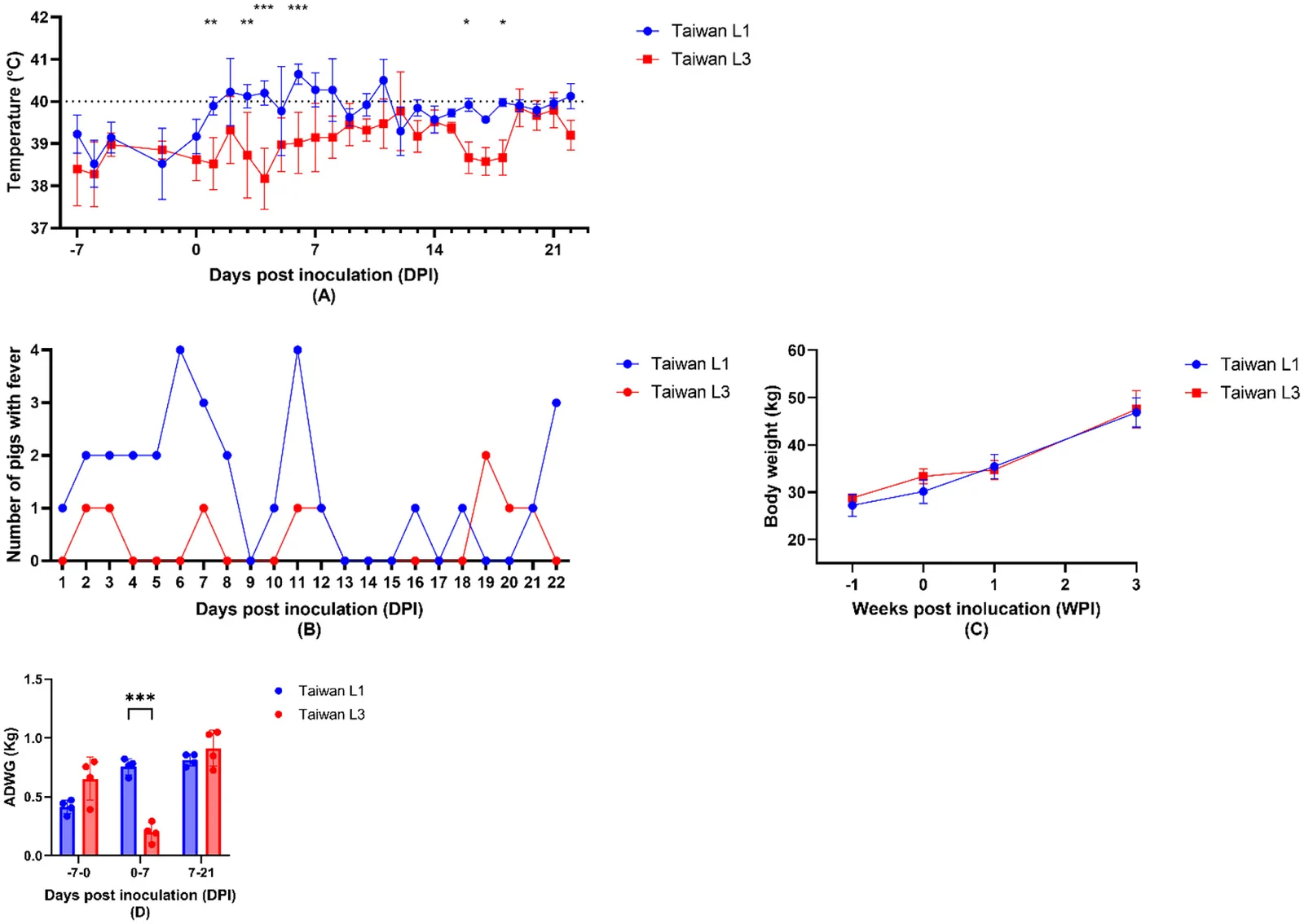
Clinical outcomes following inoculation with Taiwan PRRSV-2 L1 and L3 strains. 10-week-old pigs were inoculated intramuscularly with 1 mL containing 10^4^ TCID_50_ and intranasally with an additional 1 mL containing 10^4^ TCID_50_ of either SW2018001-YL (Taiwan L3) or NPUST-107-844/2018 (Taiwan NADC-like L1), corresponding to a total dose of 2 × 10^4^ TCID_50_ per pig. **(A)** Daily body temperature. The dotted line indicates the fever threshold of ≥40.0°C. **(B)** Number of pigs with fever at each observation time point. **(C)** Body weight during the experimental period. **(D)** Average daily weight gain during the pre-inoculation period (−7 to 0 DPI), early post-inoculation period (0–7 DPI), and later post-inoculation period (7–21 DPI). Data in A, C, and D are presented as the mean ± standard deviation. Daily body temperatures in A were analyzed using a mixed-effects model fitted by restricted maximum likelihood, followed by Šídák's multiple-comparisons test. Body weight and average daily weight gain in panels C and D were analyzed separately using two-way repeated-measures ANOVA, followed by Šídák's multiple-comparisons test. *P < 0.05, **P < 0.01, ***P < 0.001.

### 3.2 Plasma and tissue viral load quantified by RT-qPCR

PRRSV RNA was detected in plasma at 3 DPI, and the mean plasma viral RNA load peaked at 7 DPI in both groups (Figure 2A). No statistically significant difference in peak plasma viral RNA load was detected between the L1- and L3-inoculated groups. A slower decline in the mean plasma viral RNA load was observed in the L3-inoculated group than in the L1-inoculated group; however, this difference was not statistically significant.

**Figure 2:**
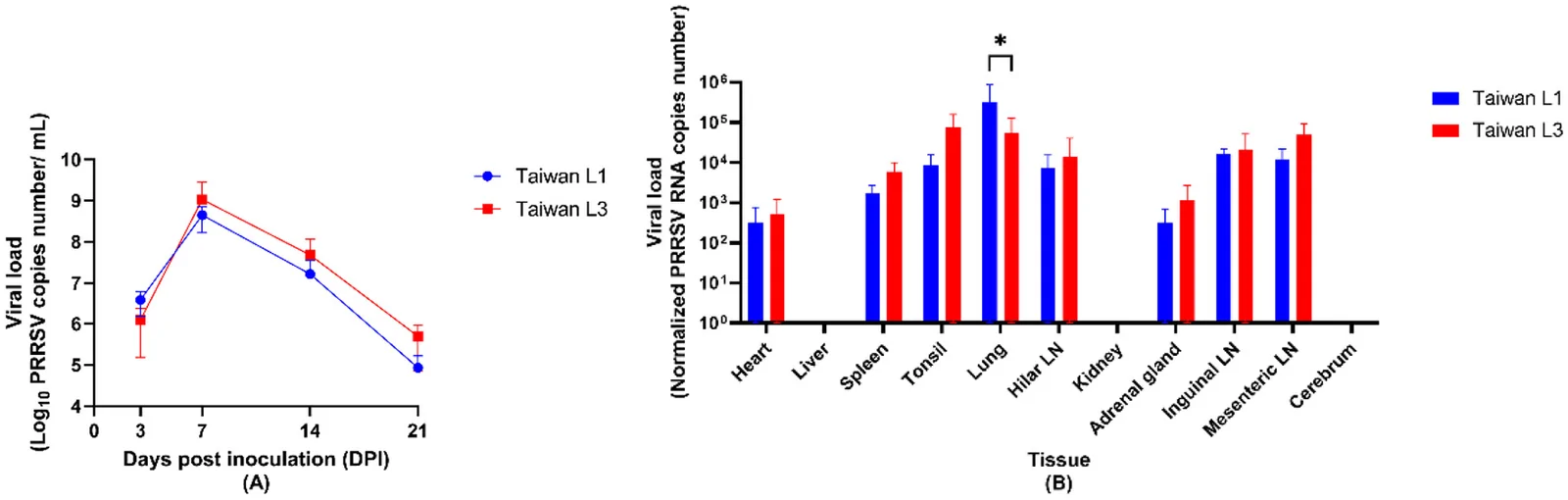
Plasma and tissue PRRSV RNA loads following inoculation with Taiwan PRRSV-2 L1 and L3 strains. Pigs were inoculated with a total dose of 2 × 10^4^ TCID_50_ of either NPUST-107-844/2018 (Taiwan L1) or SW2018001-YL (Taiwan L3) through concurrent intramuscular and intranasal administration. **(A)** Plasma PRRSV RNA loads measured at 3 DPI and weekly thereafter through 3 WPI. Results are expressed as log_10_ PRRSV RNA copies/mL of plasma. **(B)** PRRSV RNA levels in tissues collected at necropsy at 3 WPI. Tissue PRRSV RNA measurements were normalized to the corresponding porcine GAPDH transcript measurements. Data are presented as the mean ± standard deviation. *P < 0.05.

At necropsy, PRRSV RNA was detected in multiple tissues, including the heart, spleen, tonsil, adrenal gland, and lung, whereas it was not detected in the liver, kidney, or cerebrum of either group (Figure 2B). The normalized pulmonary PRRSV RNA level was significantly higher in the L1-inoculated group than in the L3-inoculated group (*P* < 0.05). No statistically significant between-group differences were detected in the remaining tissues.

### 3.3 Pathological examination and immunohistochemistry staining

At necropsy, the lungs of pigs in both inoculation groups showed diffuse tan-to-red mottling, irregularly demarcated areas, and consolidation (Figures 3A, D). Enlargement of the inguinal and hilar lymph nodes was also observed. The mean gross lung lesion scores were 72.50 ± 11.73% and 81.25 ± 17.85% in the L1- and L3-inoculated groups, respectively, with no statistically significant between-group difference (Figure 3G).

**Figure 3:**
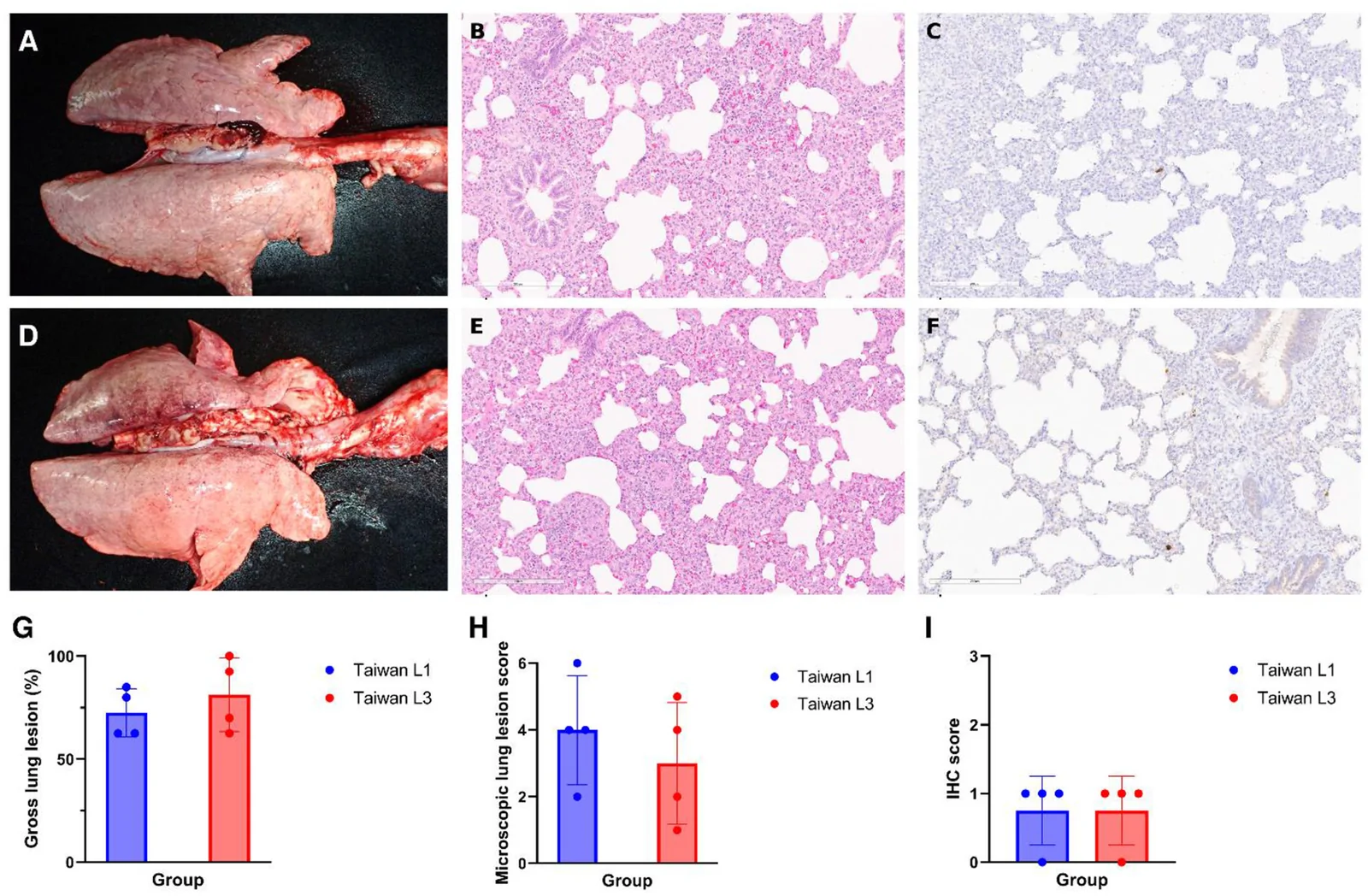
Pathological evaluation and PRRSV antigen detection in pigs. All pigs were euthanized at 3 WPI for pathological and immunohistochemical examination. Representative gross lung lesions from the **(A)** L1- and **(D)** L3-inoculated groups. Representative pulmonary histopathological findings in the **(B)** L1- and **(E)** L3-inoculated groups (200X; bar = 200 μm). Representative immunohistochemical staining for PRRSV N protein in the lungs of the **(C)** L1- and **(F)** L3-inoculated groups (200X; bar = 200 μm). Scores for **(G)** gross lung lesions, **(H)** microscopic lung lesions, and **(I)** PRRSV immunoreactivity. Data are presented as the mean ± standard deviation.

Histologically, multifocal-to-coalescing interstitial pneumonia was observed in both groups (Figures 3B, E). The mean microscopic lung lesion scores were 4.00 ± 1.63 and 3.00 ± 1.83 in the L1- and L3-inoculated groups, respectively; the difference was not statistically significant (Figure 3H).

Immunohistochemical staining demonstrated PRRSV antigen-positive cells, predominantly alveolar macrophages and mononuclear cells within the pulmonary interstitium, in both groups (Figures 3C, F). The IHC scores were 0.75 ± 0.50 in both the L1- and L3-inoculated groups, with no statistically significant between-group difference (Figure 3I).

### 3.4 Humoral and cellular immune response

Both groups were negative for PRRSV-specific antibody at 0 WPI. At 1 WPI, some pigs in both groups started to present PRRSV-specific antibody. At 2 WPI, pigs in both groups showed similar levels of PRRSV-specific antibody and persisted to 3 WPI (Figure 4).

**Figure 4:**
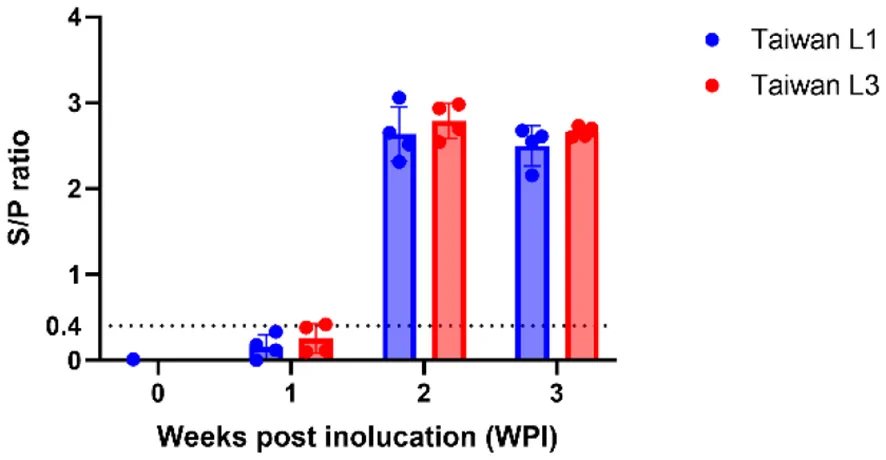
PRRSV-specific plasma antibody responses following viral inoculation. PRRSV-specific antibodies were measured using the IDEXX PRRS X3 Ab Test and expressed as sample-to-positive (S/P) ratios. An S/P ratio of ≥0.4 was considered positive. Data are presented as the mean ± standard deviation.

Following ex vivo stimulation with either the L1 or L3 strain, the L3-inoculated group showed higher mean numbers of PRRSV-specific IFN-γ-secreting cells at 1 WPI than the L1-inoculated group. The responses subsequently declined in both groups at 3 WPI (Figures 5A, B).

**Figure 5:**
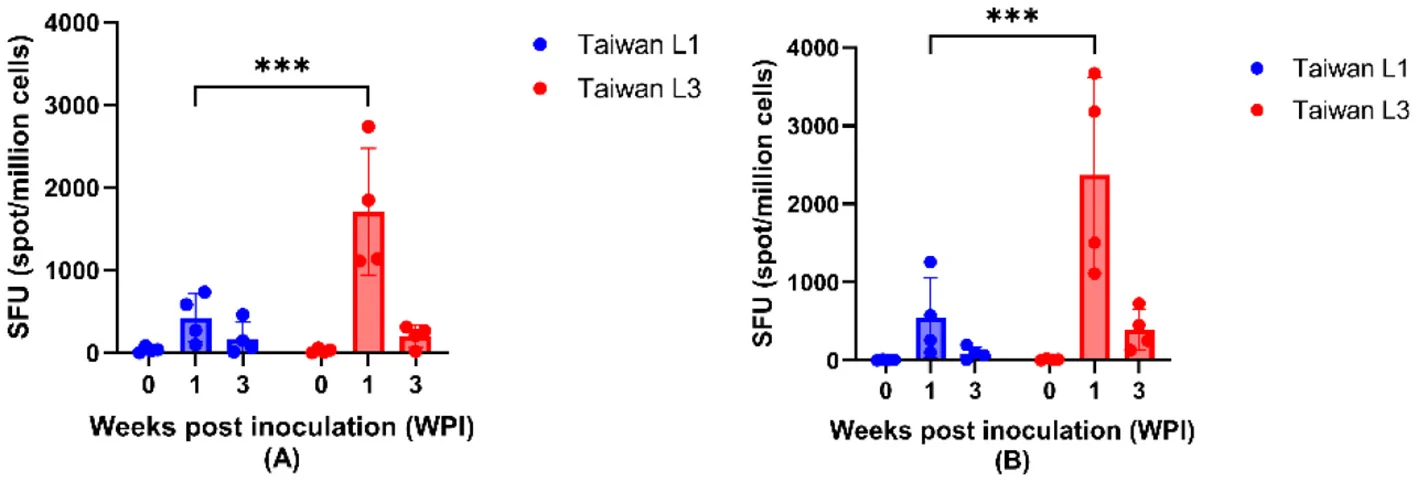
PRRSV-specific IFN-γ-secreting cell responses following viral inoculation. Peripheral blood mononuclear cells were stimulated ex vivo with **(A)** NPUST-107-844/2018 (Taiwan NADC-like L1) or **(B)** SW2018001-YL (Taiwan L3). Responses are expressed as spot-forming units per 10^6^ PBMCs. Data are presented as the mean ± standard deviation. Differences were analyzed using two-way repeated-measures ANOVA with inoculation group and time as factors, followed by Šídák's multiple-comparisons test. ***P < 0.001.

### 3.5 Cytokine response in plasma following virus inoculation

Transient increases in the mean plasma concentrations of IL-1β and IL-4 were observed in the L1-inoculated group at 1 WPI, followed by a decline from 2 WPI onward (Figures 6A, B). The L3-inoculated group showed lower mean IL-1β and IL-4 concentrations during the corresponding period. Conversely, higher mean TNF-α concentrations at 2 WPI and IL-10 concentrations at 2–3 WPI were observed in the L3-inoculated group (Figures 6C, D). However, none of the measured plasma cytokine concentrations differed significantly between the two inoculation groups.

**Figure 6:**
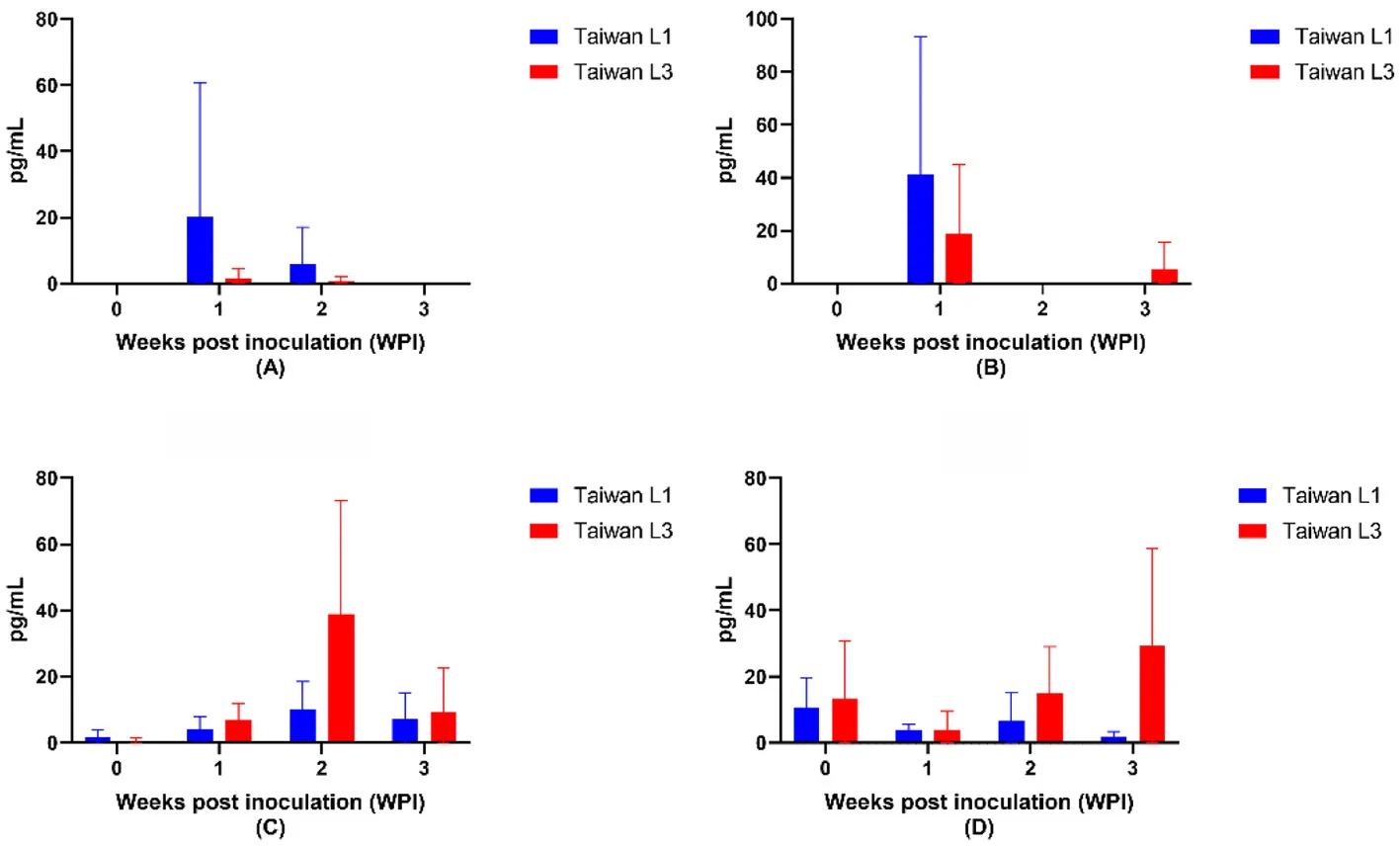
Plasma cytokine concentrations following inoculation with Taiwan PRRSV-2 L1 and L3 strains. Plasma concentrations of **(A)** IL-1β, **(B)** IL-4, **(C)** TNF-α, and **(D)** IL-10 were measured using commercial ELISA assays. Data are expressed in pg/mL and presented as the mean ± standard deviation. No statistically significant between-group differences were detected.

## 4 Discussion

The emergence of NADC30/34-like strains has been reported in China since 2013, in South Korea since 2014, in Taiwan since 2018, and in Japan since 2024 (11, 23–25). The increasing prevalence of NADC30/34-like strains across East Asia highlights their growing importance in the field. In the present study, both Taiwan NADC30/34-like L1 and L3 isolates induced interstitial pneumonia in 10-week-old pigs, and no statistically significant between-group difference in microscopic lung lesion scores was detected. However, in terms of clinical symptoms, the Taiwan NADC30/34-like L1 induced a noticeable fever response, whereas pigs infected with the Taiwan L3 exhibited minimal fever. Additionally, plasma analysis of cytokines suggests that the Taiwan NADC30/34-like L1-induced fever may be related to, at least partially, the early induction of IL-1β and IL-4. This study not only demonstrates the distinct clinical and pathological features of two prevalent PRRSV strains in Taiwan but also establishes an experimental infection model using 10-week-old growing pigs. This model may serve as a useful experimental platform for evaluating vaccine efficacy and antiviral agents.

Previous pathogenicity studies in PRRSV-2 NADC30/34 have demonstrated that there is high diversity between different strains while viremia was successfully induced, and clinical signs, including lethargy, reduced appetite, decreased average daily weight gain, and interstitial pneumonia, were observed (26–32). Most L1 strains caused fever within a few days post-inoculation, while some L3 strains also induced fever, albeit slightly later than L1 (31, 32). In another study in Taiwan, a highly virulent L3 strain caused severe fever and mortality in 4-week-old pigs (32). The severity of interstitial pneumonia varied among PRRSV-2 lineages. Consistent with our results, NADC30-like and QYYZ-like strains in South China caused comparable lung lesions (32). These findings underscore the need for strain-specific evaluation and control strategies.

In this study, although no statistical differences were detected, the two PRRSV strains showed different temporal patterns of cytokine responses cytokine responses in pigs. Pigs infected with the Taiwan NADC-like L1 virus showed an increase in IL-1β and IL-4 as early as the first week post-inoculation, may be consistent with early proinflammatory and Th2-skewed immune response. IL-1β, a key endogenous pyrogen, is known to mediate fever through hypothalamic regulation and amplification of inflammatory cascades (33, 34), and its early upregulation may partially explain the increased febrile response observed in this group. Concurrent elevation of IL-4 indicates activation of humoral immune pathways (35, 36), which may influence viral persistence and immune modulation. The combined cytokine profile has been suggested could trigger a more intense early immune activation, potentially contributing to enhanced clinical severity during PRRSV infection (37, 38).

In contrast, pigs infected with the Taiwan L3 virus showed only mild proinflammatory cytokine responses in the early stage but an upward trend of IL-10 and TNF-α at the later stages. The observed increase in IL-10 may be consistent with anti-inflammatory regulation of inflammatory signaling pathways, potentially affecting TNF-α-associated responses and fever regulation (39, 40). Notably, in both viral-inoculated groups, similar levels of tissue pathology were noted. Whether the relatively limited early alteration of these key cytokines is associated with the absence of fever in L3-infected pigs remains to be determined. (41, 42) However, given the lack of statistically significant differences, these findings should be regarded as exploratory and cannot establish a causal relationship between cytokine responses and clinical manifestations.

This study demonstrates the pathogenicity comparison of the Taiwan L1 and L3 PRRSV-2 isolates. Except for the difference in the frequency of fever, both viruses caused severe interstitial pneumonia in growing pigs in the present study, and no statistically significant differences were detected between the two isolates for several measured pathogenicity parameters. Several considerations should be taken into account when interpreting these findings. The two PRRSV isolates were propagated in different cell types according to their respective growth characteristics, which may potentially influence viral phenotypes. To minimize potential cell-adaptation effects, low-passage (P6) virus stocks were used for challenge. Although an uninfected control group was not included, both groups had comparable pre-challenge clinical and virological baselines, and active PRRSV infection was supported by consistent clinical, virological, serological, and pathological findings. Despite the relatively small sample size, the study demonstrated distinct patterns of pathogenicity between the two isolates. The findings provide useful comparative information on these Taiwan PRRSV isolates, and both isolates may serve as challenge viruses in growing pigs for future experimental studies, including vaccine efficacy evaluation, using the growing-pig infection model as a practical platform.

## Data Availability

The raw data supporting the conclusions of this article will be made available by the authors, without undue reservation.
